# The Challenges in Treating Inflammatory Bowel Diseases During the COVID-19 Pandemic: An Opinion

**DOI:** 10.3390/jcm13237128

**Published:** 2024-11-25

**Authors:** Jonatan Vukovic, Ivana Jukic, Ante Tonkic

**Affiliations:** 1 Department of Gastroenterology and Hepatology, Internal Clinic, Clinical Hospital Centre Split, Spinciceva 1, 21000 Split, Croatia; jonatan.vukovic@mefst.hr (J.V.);; 2 Department of Internal Medicine, School of Medicine, University of Split, 21000 Split, Croatia; 3 University Department of Health Studies, University of Split, 21000 Split, Croatia

**Keywords:** inflammatory bowel diseases, COVID-19, pandemic, Crohn’s disease, ulcerative colitis, treatment, SARS-CoV-2 infection, healthcare challenges

## Abstract

The COVID-19 pandemic posed significant challenges in the treatment of chronic diseases, particularly inflammatory bowel diseases (IBDs) such as Crohn’s disease and ulcerative colitis. These challenges are multifaceted, encompassing difficulties in maintaining routine care, concerns about the safety of immunosuppressive therapies, disruptions in healthcare delivery, and the complexities of managing IBD in patients who contract COVID-19. This article explores the various obstacles faced in the treatment of IBD during the pandemic and discusses potential strategies to overcome these challenges.

## 1. Introduction

Inflammatory bowel disease (IBD) is a chronic, relapsing inflammatory condition of the gastrointestinal tract, encompassing Crohn’s disease, ulcerative colitis, and other related disorders [1]. The hallmark of IBD is inflammation of the intestinal mucosa, characterized by episodes of abdominal pain, diarrhea, hematochezia, weight loss, and a marked infiltration of neutrophils and macrophages. These immune cells release cytokines, proteolytic enzymes, and reactive oxygen species, leading to tissue inflammation and ulceration [1,2]. IBD typically manifests early in life, affecting both males and females, and persists throughout the patient’s lifetime. IBD development is influenced by a complex interplay of genetic, environmental, and immune-related factors. Genetic predispositions play a significant role, as individuals with a family history of IBD are at higher risk. Environmental triggers, such as diet, smoking, and antibiotic use, can disrupt gut microbiota, further contributing to IBD onset. Additionally, immune dysregulation, where the body’s immune system mistakenly attacks the intestinal lining, is central to disease pathogenesis. Common comorbidities associated with IBD include conditions like arthritis, skin disorders, and liver diseases, highlighting the systemic nature of this chronic inflammatory disease [3].

The incidence and prevalence of IBD have significantly risen since the mid-20th century, establishing it as one of the most prevalent gastrointestinal disorders by the early 21st century, with a rapidly increasing incidence in newly industrialized countries [4,5,6]. The highest prevalence rates of IBD have been reported in Europe—ulcerative colitis at 505 per 100,000 individuals in southeastern Norway, and Crohn’s disease at 322 per 100,000 in Hesse, Germany—and in North America, where ulcerative colitis and Crohn’s disease were reported at 286.3 and 318.5 per 100,000 individuals, respectively, in Olmsted County, USA, and Nova Scotia, Canada [6]. Since 1990, the incidence of IBD in Western countries has either stabilized or begun to decline. In contrast, the incidence in newly industrialized regions of Asia, Africa, and South America has shown a marked increase [6]. The burden of IBD in the elderly population is expected to rise significantly by 2051, despite declining of the age-standardized incidence rate, highlighting the need for targeted healthcare strategies and resources worldwide [7].

Crohn’s disease most commonly affects the terminal ileum, cecum, perianal region, and colon; although, it can involve any part of the gastrointestinal tract in a discontinuous manner [3,8,9]. Ulcerative colitis, on the other hand, characteristically involves the rectum and can extend to affect part or the entirety of the colon in a continuous fashion [3,8,9]. Histologically, Crohn’s disease is distinguished by thickening of the submucosa, transmural inflammation, fissuring ulcers, and the presence of granulomas. In contrast, ulcerative colitis is characterized by inflammation confined to the mucosa and submucosa, with features such as cryptitis and crypt abscesses [3,9,10]. Inflammatory bowel diseases are chronic, immune-mediated conditions that require lifelong management. Treatment typically involves the use of immunosuppressive therapies to control inflammation and prevent disease flares.

A key component of managing IBD is regular monitoring through colonoscopy, which is vital for assessing the extent and severity of mucosal inflammation, detecting dysplasia, and guiding therapeutic decisions. Colonoscopy not only aids in confirming the diagnosis but also plays a critical role in the ongoing surveillance for colorectal cancer, a known complication of long-standing IBD. The advent of the COVID-19 pandemic had precipitated an array of unprecedented challenges in the management of IBD, fundamentally altering the landscape of care for these patients. The global health crisis prompted widespread disruptions in healthcare delivery, severely impacting access to critical services for patients with IBD. Routine procedures, including colonoscopies and other endoscopic evaluations crucial for disease monitoring and management, were often deferred or canceled, leading to significant delays in diagnosis and treatment modifications. These interruptions in care heightened the risk of disease exacerbations and complications, underscoring the vulnerability of patients with IBD in the face of systemic healthcare disruptions.

Simultaneously, the reliance on immunosuppressive therapies, essential for controlling the chronic inflammation characteristic of IBD, introduced complex dilemmas in the context of the pandemic. The immunosuppressive nature of these therapies, while necessary for maintaining disease remission, posed a potential risk of increased susceptibility to severe COVID-19 outcomes. This risk created a significant clinical conundrum, as healthcare providers had to balance the continuation of these critical therapies against the potential dangers posed by COVID-19, often in the absence of robust data to guide decision-making. The uncertainty surrounding the impact of immunosuppressive drugs on COVID-19 outcomes, particularly in the early stages of the pandemic, led to considerable anxiety and hesitation among both patients and clinicians.

Moreover, the rapid transition to telemedicine as a primary mode of healthcare delivery, although a necessary adaptation, presented additional challenges. While telemedicine offered a vital lifeline for maintaining continuity of care, it also highlighted inherent limitations, particularly for patients requiring physical examinations or in-person procedures. The lack of physical interaction posed difficulties in accurately assessing disease activity and responding to acute flares, further complicating the management of IBD during the pandemic. Additionally, the psychological burden of living with a chronic disease like IBD was exacerbated by the pandemic, as patients faced heightened anxiety over their vulnerability to COVID-19, compounded by the stress of navigating an altered healthcare environment.

The introduction of COVID-19 vaccines offered a promising avenue for mitigating the risks posed by the virus; however, it also introduced new complexities in the management of IBD. Patients on immunosuppressive therapies faced uncertainties regarding vaccine efficacy and safety, prompting the need for individualized vaccination strategies. The timing of vaccination in relation to immunosuppressive treatment schedules became a critical consideration, with the potential need for booster doses to ensure adequate immune protection, further complicating care protocols.

The COVID-19 pandemic had profoundly impacted the management of IBD, presenting a unique set of challenges that have necessitated rapid adaptations in clinical practice. These challenges have underscored the need for a flexible, evidence-based approach to IBD care, capable of responding to the evolving landscape of the pandemic while ensuring that patients receive the comprehensive care necessary to manage their chronic condition effectively.

The COVID-19 pandemic (official duration from March 2020 to May 2023 according to the World Health Organization) had caused serious social and economic disruptions globally, and the consequences for human health regarding the SARS-CoV-2 infection itself and numerous other diseases are still unknown [3,10]. The onset of the COVID-19 pandemic introduced unprecedented challenges to the management of IBD, raising concerns about the safety and efficacy of ongoing treatments, access to healthcare, and the risk of severe outcomes in patients who contract COVID-19. This manuscript aims to explore the challenges and adaptations required in IBD management during the COVID-19 pandemic, highlighting the need for an adaptable, evidence-based approach to ensure comprehensive patient care amidst evolving healthcare demands.

## 2. Impact of COVID-19 on Routine IBD Care

### 2.1. Disruptions in Healthcare Access

One of the most immediate challenges posed by the COVID-19 pandemic was the disruption of routine healthcare services. Lockdowns, social distancing measures, and the reallocation of healthcare resources to manage COVID-19 patients resulted in the postponement or cancellation of many non-urgent medical appointments, including regular follow-ups for patients with IBD. Endoscopic procedures, which are critical for diagnosing and monitoring IBD, were particularly impacted, leading to delays in new IBD diagnosis and treatment adjustments [11]. Additionally, the results from the new retrospective study have shown a reduction in both elective and emergency IBD operations during the COVID-19 pandemic [12].

These disruptions have had significant consequences for patients with IBD, who rely on regular monitoring and timely interventions to manage their condition effectively. The lack of access to healthcare has increased the risk of disease flares, complications, and in some cases, hospitalizations, which in turn place additional strain on healthcare systems already burdened by COVID-19 [11,13].

### 2.2. Delays in Diagnosis and Treatment Initiation

For individuals experiencing new or worsening IBD symptoms during the pandemic, delays in seeking medical attention have been a common issue. Fear of contracting COVID-19 in healthcare settings has led some patients to postpone visits to their healthcare providers. This has resulted in delays in the diagnosis of new IBD cases and the initiation of appropriate treatment, potentially leading to more severe disease progression [13,14].

Moreover, the reduction in available endoscopy slots and the hesitation of patients to undergo invasive procedures during the pandemic have further contributed to delays in diagnosis. In many cases, healthcare providers have had to rely more heavily on non-invasive markers of inflammation, such as fecal calprotectin and C-reactive protein (CRP) levels, to guide treatment decisions in the absence of endoscopic confirmation [14]. The effects of the pandemic on non-COVID-19 health outcomes, especially for chronic diseases, may take years to become fully evident and should continue to be an area of study, according to the research from 2021 [15].

## 3. Challenges in Immunosuppressive Treatment

### 3.1. Concerns About Immunosuppressive Therapy and COVID-19 Risk

A major concern during the pandemic has been the safety of continuing immunosuppressive therapies in patients with IBD. These therapies, which include corticosteroids, thiopurines, methotrexate, and biologics, are essential for controlling inflammation and maintaining remission in patients with IBD. However, they also suppress the immune system, potentially increasing the risk of infections, including COVID-19 [16].

Early in the pandemic, there was considerable uncertainty about whether patients on immunosuppressive therapies were at increased risk of contracting COVID-19 or experiencing more severe disease. This uncertainty led to difficult decisions for both patients and healthcare providers regarding the continuation, modification, or temporary cessation of treatment. In some cases, patients independently chose to discontinue or reduce their medications out of fear, which led to disease flares and complications.

Over time, data from registries such as SECURE-IBD provided some reassurance, indicating that while certain therapies, such as corticosteroids, are associated with worse COVID-19 outcomes, other treatments, particularly biologics, do not significantly increase the risk of severe COVID-19 [16]. Nevertheless, the management of immunosuppressive therapy during the pandemic remains complex, requiring careful consideration of the risks and benefits on a case-by-case basis.

COVID-19 infection can trigger a hyperinflammatory response, known as a “cytokine storm”, characterized by a surge of pro-inflammatory cytokines like IL-6, TNF-α, and IL-1β. This intense immune reaction is associated with severe COVID-19 outcomes, including acute respiratory distress syndrome, multiorgan failure, and increased mortality. For patients with inflammatory bowel disease (IBD), who already experience immune dysregulation and chronic inflammation, the cytokine storm poses additional risks, potentially intensifying gastrointestinal symptoms and increasing the likelihood of systemic complications.

IBD treatments, such as corticosteroids, thiopurines, and JAK inhibitors, aim to control inflammation but also suppress immune function, potentially elevating the risk of severe COVID-19. Broad immunosuppressants like corticosteroids are associated with worse COVID-19 outcomes due to their extensive immunosuppressive effects, which can impair viral clearance and increase vulnerability to infections. In contrast, targeted therapies like anti-TNF agents may be safer during COVID-19, as they can modulate cytokine activity with less systemic immune suppression.

This complex interplay underscores the need for personalized treatment approaches in patients with IBD who contract COVID-19. In mild cases, IBD treatment may be continued with careful monitoring. However, in moderate-to-severe COVID-19 cases, adjustments—especially in corticosteroid regimens—may be needed to balance the risks of exacerbating the cytokine storm against the need to manage IBD symptoms. This approach highlights the importance of understanding the inflammatory pathways shared by COVID-19 and IBD to optimize patient outcomes through adaptive management strategies [17]. Zhang C. et al. concluded that COVID-19 has potential to exacerbate the progression of IBD via cytokine storms [18].

### 3.2. Vaccination and Immunosuppressive Therapy

The rollout of COVID-19 vaccines introduced another layer of complexity to the management of IBD during the pandemic. While vaccination is strongly recommended for patients with IBD, those patients on immunosuppressive therapies may have a reduced immune response to vaccines. This has led to concerns about the adequacy of vaccine-induced protection in these patients and the need for additional vaccine doses or boosters [19].

Healthcare providers have had to navigate the timing of vaccination in relation to IBD treatment schedules. For instance, it is often recommended to avoid administering live vaccines to patients on certain immunosuppressive therapies, and for inactivated vaccines, timing the vaccine administration in a way that maximizes immune response (e.g., spacing out the vaccine from the dose of an immunosuppressive drug) has been suggested [19].

## 4. Managing IBD in Patients with COVID-19

### 4.1. Adjusting Treatment During COVID-19 Infection

Managing IBD in patients who contract COVID-19 infection presents unique challenges. According to the result of a meta-analysis, the prevalence of COVID-19 infection in patients with IBD was 1.0% [16]. The course of COVID-19 infection in patients with IBD can vary, and the presence of an underlying inflammatory condition may complicate management. In patients with mild COVID-19 infection, continuation of IBD therapy is often recommended, with some adjustments based on the specific therapy and the severity of COVID-19 disease. For instance, corticosteroids may be tapered or discontinued, if possible, due to their association with worse COVID-19 outcomes [16].

In patients with moderate-to-severe COVID-19, more significant adjustments to IBD treatment may be necessary. This can include holding certain immunosuppressive therapies temporarily until the patient recovers from the infection [20,21]. The decision to adjust therapy must be balanced against the risk of triggering a disease flare, which could further complicate the patient’s clinical course [21].

### 4.2. Hospitalization and Intensive Care Considerations

For patients with IBD who require hospitalization for severe COVID-19 infection, managing both conditions simultaneously is particularly challenging. These patients may require intensive care measures for respiratory support, while also needing close monitoring and treatment for their IBD [22]. The use of certain medications, such as tocilizumab, an interleukin-6 inhibitor used in severe COVID-19 cases, may have implications for IBD management, as it could potentially influence intestinal inflammation [23].

Additionally, patients with IBD who are hospitalized with COVID-19 infection are at increased risk of venous thromboembolism (VTE), given the pro-inflammatory and hypercoagulable states associated with both conditions [22]. Prophylactic anticoagulation may be considered, though it must be carefully weighed against the risk of gastrointestinal bleeding, which can be a concern in IBD [22].

## 5. Strategies to Overcome Treatment Challenges

### 5.1. Telemedicine as a Solution

The rapid adoption of telemedicine during the COVID-19 pandemic had provided a crucial means of maintaining continuity of care for patients with IBD [24]. Telemedicine allows patients to receive medical consultations, discuss symptoms, and adjust treatment plans without the need for in-person visits, thus reducing the risk of exposure to SARS-CoV-2. It has also facilitated more frequent check-ins, enabling healthcare providers to monitor patients more closely during this uncertain time [24].

However, telemedicine is not without its limitations. It may not be suitable for all patients, particularly those with severe disease or those requiring procedures like an endoscopy. Additionally, the lack of physical examination and the reliance on patient-reported symptoms can pose challenges in accurately assessing disease activity.

### 5.2. Patient Education and Support

Education and support are essential components of managing IBD during the pandemic. Patients need clear, evidence-based information about the risks and benefits of continuing their treatment, the importance of vaccination, and how to recognize symptoms of both IBD flares and COVID-19 infection. Healthcare providers play a key role in addressing patients’ concerns, correcting misinformation, and providing reassurance.

Support networks, including IBD patient organizations and online communities, have been valuable resources for patients during the pandemic. These networks offer a platform for sharing experiences, accessing reliable information, and finding emotional support, which is particularly important given the heightened anxiety and uncertainty caused by the pandemic.

## 6. Conclusions

The COVID-19 pandemic had posed unique challenges for managing chronic diseases, including inflammatory bowel disease (IBD). For patients with IBD, balancing the need for ongoing immunosuppressive therapy against the heightened risk of severe COVID-19 infection has been critical. Additionally, disruptions in healthcare services and delays in accessing necessary care have impacted patient outcomes, while questions surrounding vaccination safety have further complicated care for this vulnerable population.

One of the most important developments during the pandemic had been the reliance on real-time data to guide clinical decisions. Registries like SECURE-IBD have provided invaluable insights, allowing for evidence-based recommendations that address the safety of biologic therapies and identify factors that increase the risk of severe COVID-19 outcomes in patients with IBD. These initiatives emphasize the value of global collaboration and data sharing for managing public health crises effectively.

Furthermore, the pandemic has accelerated the adoption of telemedicine, which, although initially a stopgap measure, has proven to be an effective tool for maintaining continuity of care for patients with IBD. Telemedicine has not only reduced the risk of exposure to the virus but has also provided a more flexible approach to managing chronic conditions, allowing for more frequent and accessible follow-ups. As healthcare systems move forward, the integration of telemedicine into standard IBD care may enhance patient outcomes and satisfaction, even beyond the pandemic.

The pandemic has also highlighted the importance of educating and empowering patients with IBD. Ensuring that patients understand the risks and benefits of their treatments, the importance of medication adherence, and the value of vaccination has become more essential than ever. Moreover, the need for comprehensive support networks—both medical and psychosocial—has been underscored, emphasizing a holistic approach to managing chronic diseases that addresses both physical and mental health.

Looking forward, lessons from the pandemic will likely shape IBD management in lasting ways. Continued research is crucial to understand the long-term impacts of COVID-19 on IBD, including potential shifts in disease activity, the durability of vaccine-induced immunity, and the possibility of new-onset IBD triggered by viral infections. Additionally, addressing the psychological impacts of the pandemic on patients with IBD, who have faced increased anxiety and uncertainty, will be essential in providing comprehensive care.

The COVID-19 pandemic has also highlighted the importance of resilient and adaptable healthcare systems that can respond swiftly to emerging threats while maintaining high-quality chronic care. This experience has underscored the need for contingency planning to ensure access to essential services, maintain medication supply chains, and protect vulnerable populations.

In conclusion, the management of IBD during the COVID-19 pandemic required a dynamic and multifaceted approach. The integration of new technologies, the reliance on rapidly evolving data, and the emphasis on patient-centered care have all played vital roles in navigating this unprecedented period. As we move forward, the insights gained will not only inform future responses to public health crises but will also shape the ongoing evolution of IBD management, ensuring that patients continue to receive the highest standard of care, even in the face of uncertainty.

## Data Availability

All the publications referenced in this article are publicly available on PubMed, ensuring accessibility for verification and further study. The authors have exclusively cited peer-reviewed and reputable sources to support the perspectives presented in this manuscript.
